# 3-(4-Fluoro­benzo­yl)-4-(4-fluoro­phen­yl)-4-hy­droxy-2,6-di­phenyl­cyclo­hexane-1,1-dicarbo­nitrile

**DOI:** 10.1107/S1600536814012197

**Published:** 2014-05-31

**Authors:** B. Narayana, M Sapnakumari, Balladka K. Sarojini, Jerry P. Jasinski

**Affiliations:** a Department of Studies in Chemistry, Mangalore University, Mangalagangotri 574 199, India; b Department of Studies in Chemistry, Industrial Chemistry Section, Mangalore University, Mangalagangotri 574 199, India; c Department of Chemistry, Keene State College, 229 Main Street, Keene, NH 03435-2001, USA

## Abstract

In the title compound, C_33_H_24_F_2_N_2_O_2_, the cyclo­hexane ring adopts a slightly distorted chair conformation. The dihedral angle between the planes of the phenyl rings is 71.80 (9)°, while the planes of the fluoro­phenyl and fluoro­benzoyl rings are inclined to one another by 31.04 (10)°. The dihedral angles between the planes of the phenyl ring adjacent to the 4-hydroxy group and those of the the fluoro­phenyl and fluoro­benzoyl rings are 51.64 (10) and 34.31 (10)°, respectively, while the corresponding angles for the phenyl ring adjacent to the 3-(4-fluorobenzoyl) group are 57.51 (9) and 85.02 (10)°, respectively. An intra­molecular O—H⋯O hydrogen bond generates an *S*(6) ring motif. In the crystal, mol­ecules are linked *via* pairs of O—H⋯N hydrogen bonds, forming inversion dimers. The dimers are linked *via* C—H⋯N and C—H⋯O hydrogen bonds, forming chains along the *c*-axis direction. C—H⋯F hydrogen bonds link the chains into sheets lying parallel to the *bc* plane.

## Related literature   

For related structures, see: Sadikhova *et al.* (2011 ▶); Echeverria *et al.* (1995 ▶). For ring puckering parameters, see Cremer & Pople (1975 ▶). For standard bond lengths, see: Allen *et al.* (1987 ▶).
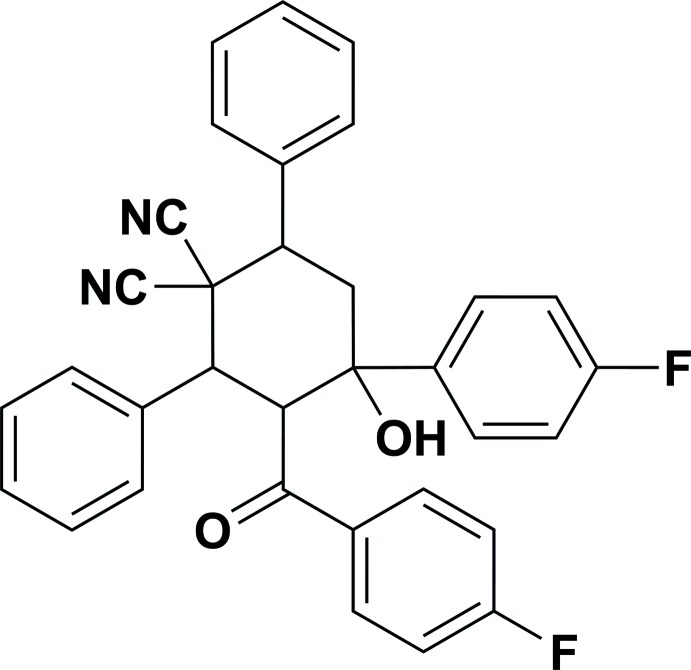


## Experimental   

### 

#### Crystal data  

C_33_H_24_F_2_N_2_O_2_
*M* *_r_* = 518.54
Triclinic, 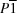

*a* = 10.9336 (10) Å
*b* = 11.5258 (4) Å
*c* = 11.8490 (7) Å
α = 89.440 (4)°
β = 62.687 (7)°
γ = 89.296 (5)°
*V* = 1326.60 (17) Å^3^
*Z* = 2
Cu *K*α radiation
μ = 0.74 mm^−1^
*T* = 173 K
0.44 × 0.32 × 0.14 mm

#### Data collection  

Agilent Eos Gemini diffractometer
Absorption correction: multi-scan (*CrysAlis PRO* and *CrysAlis RED*; Agilent, 2012 ▶) *T*_min_ = 0.884, *T*_max_ = 1.000
8616 measured reflections
5042 independent reflections
4307 reflections with *I* > 2σ(*I*)
*R*_int_ = 0.032

#### Refinement  

*R*[*F* ^2^ > 2σ(*F* ^2^)] = 0.049
*wR*(*F* ^2^) = 0.139
*S* = 1.06
5042 reflections
353 parameters
H-atom parameters constrained
Δρ_max_ = 0.26 e Å^−3^
Δρ_min_ = −0.35 e Å^−3^

### 

Data collection: *CrysAlis PRO* (Agilent, 2012 ▶); cell refinement: *CrysAlis PRO*; data reduction: *CrysAlis RED* (Agilent, 2012 ▶); program(s) used to solve structure: *SUPERFLIP* (Palatinus & Chapuis, 2007 ▶; Palatinus & van der Lee, 2008 ▶; Palatinus *et al.*, 2012 ▶).; program(s) used to refine structure: *SHELXL2012* (Sheldrick, 2008 ▶); molecular graphics: *OLEX2* (Dolomanov *et al.*, 2009 ▶); software used to prepare material for publication: *OLEX2*.

## Supplementary Material

Crystal structure: contains datablock(s) I. DOI: 10.1107/S1600536814012197/su2739sup1.cif

Structure factors: contains datablock(s) I. DOI: 10.1107/S1600536814012197/su2739Isup2.hkl

Supporting information file. DOI: 10.1107/S1600536814012197/su2739Isup3.cml

CCDC reference: 1005354

Additional supporting information:  crystallographic information; 3D view; checkCIF report

## Figures and Tables

**Table 1:** Hydrogen-bond geometry (Å, °)

*D*—H⋯*A*	*D*—H	H⋯*A*	*D*⋯*A*	*D*—H⋯*A*
O1—H1⋯O2	0.84	2.14	2.8086 (16)	136
O1—H1⋯N1^i^	0.84	2.55	3.2071 (18)	136
C15—H15⋯N2^ii^	0.95	2.55	3.388 (2)	148
C23—H23⋯O2^i^	0.95	2.49	3.394 (2)	160
C29—H29⋯O1^i^	0.95	2.49	3.398 (2)	160
C24—H24⋯F2^iii^	0.95	2.58	3.443 (2)	152

Symmetry codes: (i) 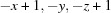; (ii) 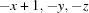; (iii) 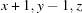.

**Fig. 1.**
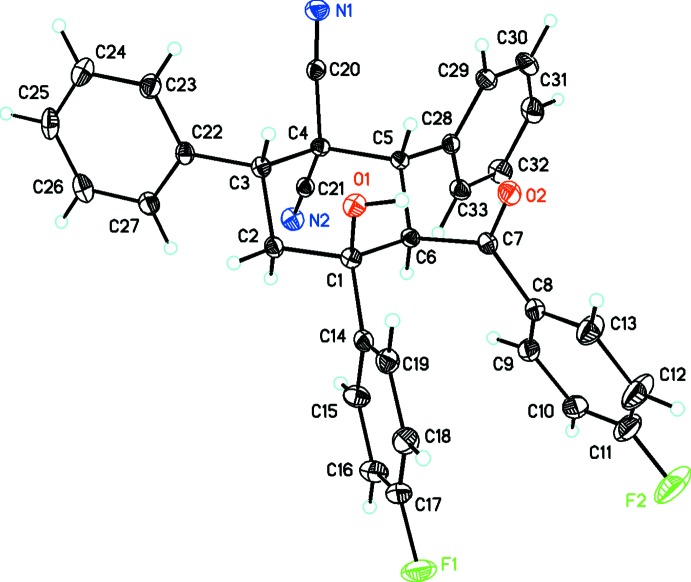
A view of the molecular structure of the title molecule, with atom labelling. Displacement ellipsoids are drawn at the 30% probability level.

**Fig. 2.**
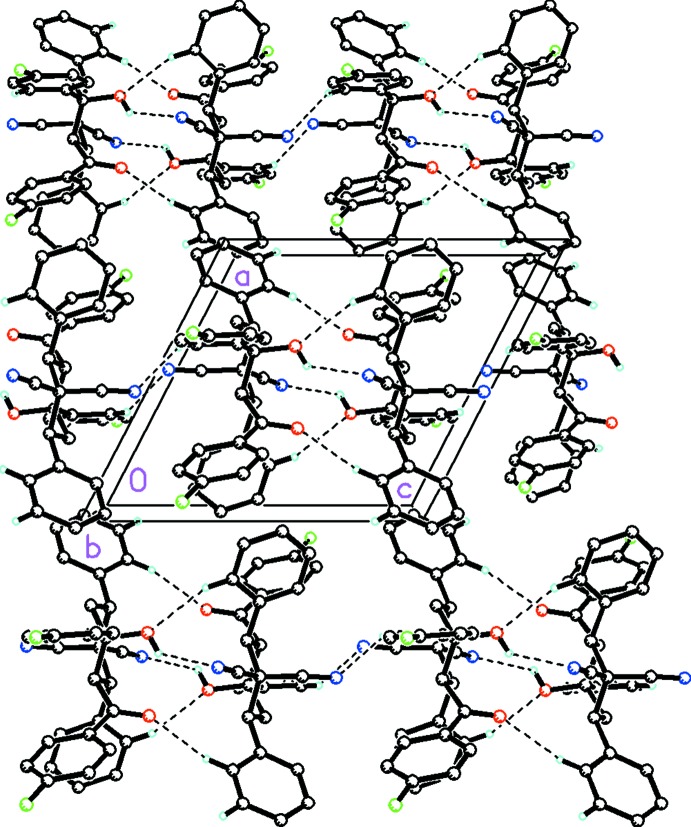
A view along along the b axis of the crystal packing of the title compound. The hydrogen bonds are shown as dashed lines (see Table 1 for details; H atoms not involved in hydrogen bonding have been omitted for clarity).

**Fig. 3.**
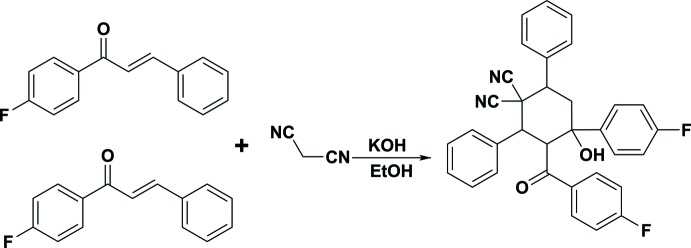
Reaction scheme.

## supplementary crystallographic information

### 1. Comment

In order to prepare the pyran derivative, (2*E*)-1-(4-fluorophenyl)-3-phenylprop-2-en-1-one was reacted with malanonitrile in the presence of a catalytic amount of ethanoic KOH. Instead of the pyran derivative, the title compound was obtained and we report herein on its crystal structure. The crystal structures of related compounds have been reported (Sadikhova *et al.*, 2011; Echeverria *et al.*, 1995).

In the molecule of the title compound, Fig. 1, the cyclohexane ring adopts a slightly distorted chair conformation [puckering parameters Q, θ, and φ = 0.5873 (17) Å, 7.19 (17)° and 50.9 (13)°, respectively; Cremer & Pople, 1975]. The dihedral angle between the phenyl rings (C22-C27 and C28-C33) is 71.80 (9)° while the fluorophenyl (C14–C19) and fluorobenzoyl (C8–C13) rings are inclined to one another by 31.04 (10)°. The dihedral angle between the phenyl ring adjacent to the 4-hydroxy group [C22–C27] and the fluorophenyl and fluorobenzoyl rings is 51.64 (10) and 34.31 (10) °, respectively, while the corresponding angles for the phenyl ring adjacent to the 3-(4-fluorobenzoyl) group [C8–C13] are 57.51 (9) and 85.02 (10) °, respectively. Bond lengths are in normal ranges (Allen *et al.*, 1987). There is an intramolecular O—H···O hydrogen bond generating an S(6) ring motif (Table 1).

In the crystal, molecules are linked via O-H···N hydrogen bonds forming inversion dimers. The dimers are linked via C-H···N and C-H···O hydrogen bonds forming chains along [001]. C-H···F hydrogen bonds link the chains to form sheets lying parallel to the bc plane (Table 1 and Fig. 2).

### 2. Experimental

A mixture of (2*E*)-1-(4-fluorophenyl)-3-phenylprop-2-en-1-one (4.52g, 0.02 mol) and malanonitrile (0.55ml, 0.01 mol) in 30 ml ethanol in the presence of a catalytic amount of ethanoic KOH was stirred at room temperature for 6 h. The precipitate obtained was collected by filtration and purified by recrystallization from ethanol. Prismatic colourless crystals were grown from ethanol by the slow evaporation method (M.p. 497–499 K).

### 3. Refinement

All of the H atoms were placed in calculated positions and refined using the riding model approximation: O-H = 0.84 Å, C-H = 0.95 - 1.00 Å with U_iso_(H) = 1.5U_eq_(O) and = 1.2U_eq_(C) for other H atoms.

### Crystal data

**Table:**

C_33_H_24_F_2_N_2_O_2_	*Z* = 2
*M**_r_* = 518.54	*F*(000) = 540
Triclinic, *P*1	*D*_x_ = 1.298 Mg m^−^^3^
*a* = 10.9336 (10) Å	Cu *K*α radiation, λ = 1.54184 Å
*b* = 11.5258 (4) Å	Cell parameters from 3787 reflections
*c* = 11.8490 (7) Å	θ = 4.2–71.1°
α = 89.440 (4)°	µ = 0.74 mm^−^^1^
β = 62.687 (7)°	*T* = 173 K
γ = 89.296 (5)°	Prism, colourless
*V* = 1326.60 (17) Å^3^	0.44 × 0.32 × 0.14 mm

### Data collection

**Table:**

Agilent Eos Gemini diffractometer	5042 independent reflections
Radiation source: Enhance (Cu) X-ray Source	4307 reflections with *I* > 2σ(*I*)
Graphite monochromator	*R*_int_ = 0.032
Detector resolution: 16.0416 pixels mm^-1^	θ_max_ = 71.4°, θ_min_ = 3.8°
ω scans	*h* = −13→13
Absorption correction: multi-scan (*CrysAlis PRO* and *CrysAlis RED*; Agilent, 2012)	*k* = −11→14
*T*_min_ = 0.884, *T*_max_ = 1.000	*l* = −14→14
8616 measured reflections	

### Refinement

**Table:**

Refinement on *F*^2^	Primary atom site location: structure-invariant direct methods
Least-squares matrix: full	Hydrogen site location: inferred from neighbouring sites
*R*[*F*^2^ > 2σ(*F*^2^)] = 0.049	H-atom parameters constrained
*wR*(*F*^2^) = 0.139	*w* = 1/[σ^2^(*F*_o_^2^) + (0.0729*P*)^2^ + 0.0921*P*] where *P* = (*F*_o_^2^ + 2*F*_c_^2^)/3
*S* = 1.06	(Δ/σ)_max_ < 0.001
5042 reflections	Δρ_max_ = 0.26 e Å^−^^3^
353 parameters	Δρ_min_ = −0.35 e Å^−^^3^
0 restraints	

### Special details

**Table:**

Geometry. All esds (except the esd in the dihedral angle between two l.s. planes) are estimated using the full covariance matrix. The cell esds are taken into account individually in the estimation of esds in distances, angles and torsion angles; correlations between esds in cell parameters are only used when they are defined by crystal symmetry. An approximate (isotropic) treatment of cell esds is used for estimating esds involving l.s. planes.

### Fractional atomic coordinates and isotropic or equivalent isotropic displacement parameters (Å^2^)

**Table:**

	*x*	*y*	*z*	*U*_iso_*/*U*_eq_	
F1	0.65588 (15)	0.61176 (9)	0.08267 (12)	0.0527 (3)	
F2	0.0999 (2)	0.57543 (13)	0.28697 (16)	0.0852 (6)	
O1	0.61907 (11)	0.13809 (9)	0.36838 (10)	0.0253 (2)	
H1	0.5515	0.1690	0.4296	0.038*	
O2	0.33370 (12)	0.17874 (10)	0.49569 (10)	0.0308 (3)	
N1	0.48277 (16)	−0.29229 (12)	0.38019 (14)	0.0352 (3)	
N2	0.53932 (15)	−0.12783 (12)	0.02494 (13)	0.0311 (3)	
C1	0.60207 (15)	0.14872 (12)	0.25657 (14)	0.0215 (3)	
C2	0.70686 (15)	0.06396 (12)	0.16162 (14)	0.0232 (3)	
H2A	0.6971	0.0643	0.0826	0.028*	
H2B	0.8011	0.0900	0.1400	0.028*	
C3	0.68724 (15)	−0.05931 (12)	0.21500 (14)	0.0218 (3)	
H3	0.6902	−0.0556	0.2980	0.026*	
C4	0.53892 (15)	−0.10161 (12)	0.24409 (13)	0.0201 (3)	
C5	0.42239 (14)	−0.01457 (12)	0.33284 (13)	0.0199 (3)	
H5	0.4222	−0.0166	0.4173	0.024*	
C6	0.45335 (15)	0.11141 (12)	0.28391 (13)	0.0201 (3)	
H6	0.4425	0.1202	0.2049	0.024*	
C7	0.35273 (15)	0.19258 (13)	0.38643 (14)	0.0226 (3)	
C8	0.28789 (16)	0.29313 (13)	0.35439 (15)	0.0257 (3)	
C9	0.26159 (17)	0.29536 (14)	0.25023 (16)	0.0298 (3)	
H9	0.2893	0.2316	0.1934	0.036*	
C10	0.1954 (2)	0.38977 (16)	0.22861 (18)	0.0383 (4)	
H10	0.1733	0.3903	0.1598	0.046*	
C11	0.1624 (3)	0.48256 (18)	0.3094 (2)	0.0505 (5)	
C12	0.1893 (3)	0.48520 (19)	0.4114 (2)	0.0634 (7)	
H12	0.1662	0.5514	0.4646	0.076*	
C13	0.2511 (2)	0.38869 (16)	0.43501 (19)	0.0432 (5)	
H13	0.2686	0.3877	0.5065	0.052*	
C14	0.62655 (15)	0.27342 (13)	0.20554 (14)	0.0236 (3)	
C15	0.62491 (18)	0.29980 (14)	0.09165 (16)	0.0305 (4)	
H15	0.6166	0.2391	0.0420	0.037*	
C16	0.63534 (19)	0.41405 (15)	0.04940 (17)	0.0362 (4)	
H16	0.6331	0.4323	−0.0280	0.043*	
C17	0.64891 (19)	0.49973 (14)	0.12255 (18)	0.0357 (4)	
C18	0.65521 (19)	0.47700 (14)	0.23296 (17)	0.0349 (4)	
H18	0.6676	0.5380	0.2800	0.042*	
C19	0.64313 (17)	0.36259 (14)	0.27536 (16)	0.0289 (3)	
H19	0.6462	0.3453	0.3526	0.035*	
C20	0.50902 (16)	−0.21300 (13)	0.31558 (14)	0.0238 (3)	
C21	0.53569 (15)	−0.11907 (12)	0.12229 (14)	0.0224 (3)	
C22	0.79960 (15)	−0.14352 (12)	0.13101 (15)	0.0233 (3)	
C23	0.84552 (17)	−0.22849 (14)	0.18758 (16)	0.0309 (4)	
H23	0.8069	−0.2322	0.2775	0.037*	
C24	0.9467 (2)	−0.30747 (17)	0.1143 (2)	0.0414 (4)	
H24	0.9772	−0.3647	0.1542	0.050*	
C25	1.00376 (18)	−0.30363 (17)	−0.01680 (19)	0.0401 (4)	
H25	1.0720	−0.3588	−0.0669	0.048*	
C26	0.96054 (18)	−0.21879 (16)	−0.07436 (17)	0.0362 (4)	
H26	1.0002	−0.2151	−0.1644	0.043*	
C27	0.85967 (17)	−0.13917 (14)	−0.00128 (16)	0.0299 (4)	
H27	0.8312	−0.0810	−0.0418	0.036*	
C28	0.28095 (15)	−0.05464 (12)	0.35494 (14)	0.0224 (3)	
C29	0.19609 (17)	−0.11291 (14)	0.46728 (15)	0.0289 (3)	
H29	0.2250	−0.1230	0.5311	0.035*	
C30	0.06992 (18)	−0.15621 (16)	0.48680 (17)	0.0364 (4)	
H30	0.0132	−0.1959	0.5636	0.044*	
C31	0.02670 (18)	−0.14177 (16)	0.39486 (18)	0.0382 (4)	
H31	−0.0592	−0.1722	0.4079	0.046*	
C32	0.10845 (19)	−0.08298 (16)	0.28384 (18)	0.0359 (4)	
H32	0.0782	−0.0726	0.2209	0.043*	
C33	0.23493 (16)	−0.03880 (14)	0.26357 (15)	0.0277 (3)	
H33	0.2901	0.0023	0.1873	0.033*	

### Atomic displacement parameters (Å^2^)

**Table:**

	*U*^11^	*U*^22^	*U*^33^	*U*^12^	*U*^13^	*U*^23^
F1	0.0756 (9)	0.0239 (5)	0.0491 (7)	−0.0014 (5)	−0.0205 (6)	0.0086 (5)
F2	0.1270 (15)	0.0601 (9)	0.0865 (11)	0.0545 (10)	−0.0655 (11)	−0.0114 (8)
O1	0.0276 (6)	0.0271 (5)	0.0248 (6)	0.0020 (4)	−0.0152 (5)	−0.0027 (4)
O2	0.0343 (6)	0.0367 (6)	0.0200 (6)	0.0061 (5)	−0.0114 (5)	−0.0030 (5)
N1	0.0416 (9)	0.0254 (7)	0.0315 (8)	0.0019 (6)	−0.0107 (7)	0.0033 (6)
N2	0.0353 (8)	0.0363 (7)	0.0242 (7)	−0.0007 (6)	−0.0158 (6)	−0.0030 (6)
C1	0.0218 (7)	0.0222 (7)	0.0214 (7)	−0.0019 (6)	−0.0105 (6)	0.0003 (6)
C2	0.0187 (7)	0.0239 (7)	0.0242 (7)	−0.0012 (5)	−0.0074 (6)	−0.0007 (6)
C3	0.0203 (7)	0.0242 (7)	0.0216 (7)	−0.0010 (5)	−0.0100 (6)	−0.0012 (6)
C4	0.0215 (7)	0.0201 (7)	0.0187 (7)	−0.0008 (5)	−0.0092 (6)	0.0003 (5)
C5	0.0193 (7)	0.0216 (7)	0.0175 (7)	0.0002 (5)	−0.0074 (6)	0.0010 (5)
C6	0.0200 (7)	0.0225 (7)	0.0174 (7)	−0.0006 (5)	−0.0083 (6)	0.0007 (5)
C7	0.0189 (7)	0.0253 (7)	0.0221 (7)	−0.0019 (5)	−0.0081 (6)	−0.0003 (6)
C8	0.0209 (7)	0.0266 (7)	0.0261 (8)	0.0009 (6)	−0.0076 (6)	0.0001 (6)
C9	0.0290 (8)	0.0316 (8)	0.0272 (8)	0.0024 (6)	−0.0117 (7)	−0.0003 (6)
C10	0.0396 (10)	0.0409 (10)	0.0353 (9)	0.0035 (8)	−0.0183 (8)	0.0073 (8)
C11	0.0601 (14)	0.0403 (10)	0.0533 (12)	0.0219 (10)	−0.0285 (11)	0.0013 (9)
C12	0.096 (2)	0.0423 (12)	0.0606 (14)	0.0374 (13)	−0.0443 (15)	−0.0228 (10)
C13	0.0575 (13)	0.0383 (10)	0.0399 (10)	0.0170 (9)	−0.0279 (10)	−0.0109 (8)
C14	0.0183 (7)	0.0238 (7)	0.0255 (8)	−0.0018 (5)	−0.0071 (6)	0.0007 (6)
C15	0.0342 (9)	0.0288 (8)	0.0308 (9)	−0.0084 (7)	−0.0166 (7)	0.0041 (7)
C16	0.0398 (10)	0.0347 (9)	0.0342 (9)	−0.0067 (7)	−0.0171 (8)	0.0102 (7)
C17	0.0368 (9)	0.0224 (8)	0.0386 (10)	−0.0018 (7)	−0.0094 (8)	0.0066 (7)
C18	0.0396 (10)	0.0235 (8)	0.0349 (9)	−0.0010 (7)	−0.0112 (8)	−0.0060 (7)
C19	0.0297 (8)	0.0269 (8)	0.0269 (8)	0.0008 (6)	−0.0102 (7)	−0.0032 (6)
C20	0.0241 (8)	0.0225 (7)	0.0238 (7)	0.0023 (6)	−0.0101 (6)	−0.0028 (6)
C21	0.0192 (7)	0.0207 (7)	0.0258 (8)	−0.0005 (5)	−0.0090 (6)	−0.0009 (6)
C22	0.0177 (7)	0.0249 (7)	0.0271 (8)	−0.0014 (6)	−0.0101 (6)	−0.0027 (6)
C23	0.0272 (8)	0.0362 (9)	0.0305 (8)	0.0049 (7)	−0.0143 (7)	−0.0017 (7)
C24	0.0358 (10)	0.0400 (10)	0.0497 (11)	0.0127 (8)	−0.0209 (9)	−0.0040 (8)
C25	0.0219 (8)	0.0429 (10)	0.0466 (11)	0.0084 (7)	−0.0079 (8)	−0.0146 (8)
C26	0.0249 (8)	0.0440 (10)	0.0314 (9)	−0.0023 (7)	−0.0055 (7)	−0.0085 (7)
C27	0.0252 (8)	0.0343 (8)	0.0269 (8)	−0.0001 (6)	−0.0090 (7)	−0.0004 (6)
C28	0.0188 (7)	0.0221 (7)	0.0235 (7)	0.0004 (5)	−0.0072 (6)	−0.0008 (6)
C29	0.0252 (8)	0.0333 (8)	0.0251 (8)	−0.0021 (6)	−0.0090 (7)	0.0038 (6)
C30	0.0235 (8)	0.0406 (9)	0.0356 (9)	−0.0096 (7)	−0.0054 (7)	0.0099 (7)
C31	0.0216 (8)	0.0444 (10)	0.0459 (11)	−0.0093 (7)	−0.0131 (8)	0.0020 (8)
C32	0.0299 (9)	0.0445 (10)	0.0393 (10)	−0.0051 (7)	−0.0211 (8)	0.0027 (8)
C33	0.0232 (8)	0.0327 (8)	0.0270 (8)	−0.0044 (6)	−0.0113 (7)	0.0036 (6)

### Geometric parameters (Å, º)

**Table:**

F1—C17	1.3616 (19)	C13—H13	0.9500
F2—C11	1.352 (2)	C14—C15	1.388 (2)
O1—H1	0.8400	C14—C19	1.390 (2)
O1—C1	1.4236 (17)	C15—H15	0.9500
O2—C7	1.2224 (18)	C15—C16	1.391 (2)
N1—C20	1.138 (2)	C16—H16	0.9500
N2—C21	1.141 (2)	C16—C17	1.374 (3)
C1—C2	1.531 (2)	C17—C18	1.364 (3)
C1—C6	1.5700 (19)	C18—H18	0.9500
C1—C14	1.5320 (19)	C18—C19	1.393 (2)
C2—H2A	0.9900	C19—H19	0.9500
C2—H2B	0.9900	C22—C23	1.394 (2)
C2—C3	1.5265 (19)	C22—C27	1.395 (2)
C3—H3	1.0000	C23—H23	0.9500
C3—C4	1.5778 (19)	C23—C24	1.384 (2)
C3—C22	1.520 (2)	C24—H24	0.9500
C4—C5	1.5797 (19)	C24—C25	1.383 (3)
C4—C20	1.4858 (19)	C25—H25	0.9500
C4—C21	1.4755 (19)	C25—C26	1.384 (3)
C5—H5	1.0000	C26—H26	0.9500
C5—C6	1.5410 (18)	C26—C27	1.386 (2)
C5—C28	1.5222 (19)	C27—H27	0.9500
C6—H6	1.0000	C28—C29	1.396 (2)
C6—C7	1.5270 (19)	C28—C33	1.397 (2)
C7—C8	1.486 (2)	C29—H29	0.9500
C8—C9	1.390 (2)	C29—C30	1.388 (2)
C8—C13	1.394 (2)	C30—H30	0.9500
C9—H9	0.9500	C30—C31	1.380 (3)
C9—C10	1.386 (2)	C31—H31	0.9500
C10—H10	0.9500	C31—C32	1.381 (3)
C10—C11	1.373 (3)	C32—H32	0.9500
C11—C12	1.369 (3)	C32—C33	1.393 (2)
C12—H12	0.9500	C33—H33	0.9500
C12—C13	1.386 (3)		
			
C1—O1—H1	109.5	C12—C13—H13	119.8
O1—C1—C2	105.23 (11)	C15—C14—C1	120.12 (13)
O1—C1—C6	110.58 (12)	C15—C14—C19	119.04 (14)
O1—C1—C14	111.36 (11)	C19—C14—C1	120.74 (14)
C2—C1—C6	109.00 (11)	C14—C15—H15	119.6
C2—C1—C14	111.67 (12)	C14—C15—C16	120.78 (15)
C14—C1—C6	108.96 (11)	C16—C15—H15	119.6
C1—C2—H2A	109.3	C15—C16—H16	120.9
C1—C2—H2B	109.3	C17—C16—C15	118.26 (16)
H2A—C2—H2B	107.9	C17—C16—H16	120.9
C3—C2—C1	111.75 (12)	F1—C17—C16	118.23 (17)
C3—C2—H2A	109.3	F1—C17—C18	119.05 (16)
C3—C2—H2B	109.3	C18—C17—C16	122.72 (16)
C2—C3—H3	107.2	C17—C18—H18	120.7
C2—C3—C4	108.79 (11)	C17—C18—C19	118.67 (16)
C4—C3—H3	107.2	C19—C18—H18	120.7
C22—C3—C2	113.62 (12)	C14—C19—C18	120.49 (16)
C22—C3—H3	107.2	C14—C19—H19	119.8
C22—C3—C4	112.40 (11)	C18—C19—H19	119.8
C3—C4—C5	112.19 (11)	N1—C20—C4	173.64 (16)
C20—C4—C3	109.57 (11)	N2—C21—C4	175.91 (16)
C20—C4—C5	105.38 (11)	C23—C22—C3	119.12 (14)
C21—C4—C3	108.45 (12)	C23—C22—C27	118.28 (15)
C21—C4—C5	111.69 (11)	C27—C22—C3	122.60 (14)
C21—C4—C20	109.51 (12)	C22—C23—H23	119.6
C4—C5—H5	106.8	C24—C23—C22	120.80 (16)
C6—C5—C4	111.92 (11)	C24—C23—H23	119.6
C6—C5—H5	106.8	C23—C24—H24	119.8
C28—C5—C4	111.19 (11)	C25—C24—C23	120.40 (17)
C28—C5—H5	106.8	C25—C24—H24	119.8
C28—C5—C6	113.01 (11)	C24—C25—H25	120.3
C1—C6—H6	109.7	C24—C25—C26	119.45 (17)
C5—C6—C1	111.93 (11)	C26—C25—H25	120.3
C5—C6—H6	109.7	C25—C26—H26	119.8
C7—C6—C1	106.85 (11)	C25—C26—C27	120.32 (17)
C7—C6—C5	108.83 (11)	C27—C26—H26	119.8
C7—C6—H6	109.7	C22—C27—H27	119.6
O2—C7—C6	118.56 (13)	C26—C27—C22	120.74 (16)
O2—C7—C8	119.71 (14)	C26—C27—H27	119.6
C8—C7—C6	121.51 (13)	C29—C28—C5	119.55 (14)
C9—C8—C7	123.35 (14)	C29—C28—C33	118.54 (14)
C9—C8—C13	119.38 (15)	C33—C28—C5	121.87 (13)
C13—C8—C7	117.27 (15)	C28—C29—H29	119.6
C8—C9—H9	119.7	C30—C29—C28	120.76 (16)
C10—C9—C8	120.56 (16)	C30—C29—H29	119.6
C10—C9—H9	119.7	C29—C30—H30	119.9
C9—C10—H10	120.9	C31—C30—C29	120.13 (16)
C11—C10—C9	118.12 (17)	C31—C30—H30	119.9
C11—C10—H10	120.9	C30—C31—H31	120.0
F2—C11—C10	118.07 (19)	C30—C31—C32	119.92 (15)
F2—C11—C12	118.70 (19)	C32—C31—H31	120.0
C12—C11—C10	123.23 (17)	C31—C32—H32	119.8
C11—C12—H12	120.9	C31—C32—C33	120.39 (16)
C11—C12—C13	118.27 (19)	C33—C32—H32	119.8
C13—C12—H12	120.9	C28—C33—H33	119.9
C8—C13—H13	119.8	C32—C33—C28	120.25 (15)
C12—C13—C8	120.37 (18)	C32—C33—H33	119.9
			
F1—C17—C18—C19	177.76 (16)	C6—C7—C8—C9	29.1 (2)
F2—C11—C12—C13	178.8 (3)	C6—C7—C8—C13	−151.58 (16)
O1—C1—C2—C3	56.14 (14)	C7—C8—C9—C10	177.16 (16)
O1—C1—C6—C5	−58.60 (15)	C7—C8—C13—C12	−179.6 (2)
O1—C1—C6—C7	60.44 (14)	C8—C9—C10—C11	2.9 (3)
O1—C1—C14—C15	175.19 (13)	C9—C8—C13—C12	−0.2 (3)
O1—C1—C14—C19	−8.3 (2)	C9—C10—C11—F2	178.9 (2)
O2—C7—C8—C9	−156.39 (16)	C9—C10—C11—C12	−1.4 (4)
O2—C7—C8—C13	22.9 (2)	C10—C11—C12—C13	−0.9 (4)
C1—C2—C3—C4	61.16 (15)	C11—C12—C13—C8	1.7 (4)
C1—C2—C3—C22	−172.82 (12)	C13—C8—C9—C10	−2.2 (3)
C1—C6—C7—O2	−74.24 (16)	C14—C1—C2—C3	177.09 (11)
C1—C6—C7—C8	100.34 (15)	C14—C1—C6—C5	178.70 (11)
C1—C14—C15—C16	174.53 (15)	C14—C1—C6—C7	−62.26 (14)
C1—C14—C19—C18	−175.25 (15)	C14—C15—C16—C17	0.8 (3)
C2—C1—C6—C5	56.61 (15)	C15—C14—C19—C18	1.2 (2)
C2—C1—C6—C7	175.65 (11)	C15—C16—C17—F1	−178.49 (16)
C2—C1—C14—C15	57.88 (18)	C15—C16—C17—C18	1.3 (3)
C2—C1—C14—C19	−125.66 (15)	C16—C17—C18—C19	−2.0 (3)
C2—C3—C4—C5	−54.00 (15)	C17—C18—C19—C14	0.7 (3)
C2—C3—C4—C20	−170.68 (12)	C19—C14—C15—C16	−2.0 (2)
C2—C3—C4—C21	69.84 (14)	C20—C4—C5—C6	169.33 (11)
C2—C3—C22—C23	142.45 (14)	C20—C4—C5—C28	−63.23 (14)
C2—C3—C22—C27	−37.27 (19)	C21—C4—C5—C6	−71.85 (14)
C3—C4—C5—C6	50.17 (15)	C21—C4—C5—C28	55.59 (15)
C3—C4—C5—C28	177.60 (11)	C22—C3—C4—C5	179.27 (11)
C3—C22—C23—C24	179.36 (15)	C22—C3—C4—C20	62.59 (15)
C3—C22—C27—C26	−179.05 (14)	C22—C3—C4—C21	−56.89 (15)
C4—C3—C22—C23	−93.46 (16)	C22—C23—C24—C25	−0.3 (3)
C4—C3—C22—C27	86.82 (17)	C23—C22—C27—C26	1.2 (2)
C4—C5—C6—C1	−51.18 (15)	C23—C24—C25—C26	1.1 (3)
C4—C5—C6—C7	−169.05 (11)	C24—C25—C26—C27	−0.8 (3)
C4—C5—C28—C29	99.48 (16)	C25—C26—C27—C22	−0.4 (3)
C4—C5—C28—C33	−78.01 (17)	C27—C22—C23—C24	−0.9 (2)
C5—C6—C7—O2	46.79 (17)	C28—C5—C6—C1	−177.63 (12)
C5—C6—C7—C8	−138.62 (13)	C28—C5—C6—C7	64.50 (15)
C5—C28—C29—C30	−176.31 (15)	C28—C29—C30—C31	−0.2 (3)
C5—C28—C33—C32	176.00 (15)	C29—C28—C33—C32	−1.5 (2)
C6—C1—C2—C3	−62.48 (15)	C29—C30—C31—C32	−0.7 (3)
C6—C1—C14—C15	−62.57 (18)	C30—C31—C32—C33	0.5 (3)
C6—C1—C14—C19	113.89 (15)	C31—C32—C33—C28	0.7 (3)
C6—C5—C28—C29	−133.68 (14)	C33—C28—C29—C30	1.3 (2)
C6—C5—C28—C33	48.83 (19)		

### Hydrogen-bond geometry (Å, º)

**Table:**

*D*—H···*A*	*D*—H	H···*A*	*D*···*A*	*D*—H···*A*
O1—H1···O2	0.84	2.14	2.8086 (16)	136
O1—H1···N1^i^	0.84	2.55	3.2071 (18)	136
C15—H15···N2^ii^	0.95	2.55	3.388 (2)	148
C23—H23···O2^i^	0.95	2.49	3.394 (2)	160
C29—H29···O1^i^	0.95	2.49	3.398 (2)	160
C24—H24···F2^iii^	0.95	2.58	3.443 (2)	152

Symmetry codes: (i) −*x*+1, −*y*, −*z*+1; (ii) −*x*+1, −*y*, −*z*; (iii) *x*+1, *y*−1, *z*.
